# Discrimination, Stress, and Health Across the Life Course

**DOI:** 10.1093/geroni/igaf122.1686

**Published:** 2025-12-31

**Authors:** Roland Thorpe, Carl Hill

## Abstract

There is a paucity of research that seeks to understand why race disparities in health across the life course remain elusive. Two such explanations that have been garnering attention are stress and discrimination. The symposium includes several papers addressing the effects of discrimination and stress on health disparities across the life course. Cobb used latent class analysis to identify discrimination subgroups among Black men aged 50 and older, finding that those in the Race/Ancestry Discrimination group had significantly lower odds of hospital visits. Thomas Tobin examined the association between everyday discrimination and multimorbidity among Black Americans, revealing that the discrimination-multimorbidity association was strongest among young adults and decreased among middle-aged and older adults. Kim conducted a multi-methods study on food insecurity and food environments, finding that residing in neighborhoods with low-income, low-vehicle access, and higher low-fat milk prices was associated with a greater risk of incident dementia. Qualitatively, food environmental factors relevant to healthy food purchasing and eating—both protective against dementia. Whilby characterized racial discrimination trajectories and identified correlates of the trajectories among Black adults, identifying individuals reporting higher levels of major lifetime experiences of discrimination and greater neighborhood social cohesion were associated with membership in the “moderate” and the “persistently high and increasing” racial discrimination trajectory groups. These findings highlight the variability in the cumulative patterning of racial discrimination and stress among midlife and older Black adults, providing insights into how these factors impact health outcomes in middle to late life.

